# Can-Pain-a digital intervention to optimise cancer pain control in the community: development and feasibility testing

**DOI:** 10.1007/s00520-020-05510-0

**Published:** 2020-05-28

**Authors:** Rosalind Adam, Christine M. Bond, Christopher D. Burton, Marijn de Bruin, Peter Murchie

**Affiliations:** 1 Academic Primary Care, Institute of Applied Health Sciences, University of Aberdeen, Room 1:020, Polwarth Building, Foresterhill, Aberdeen, AB25 2ZD UK; 2 Academic Primary Care, Institute of Applied Health Sciences, University of Aberdeen, Aberdeen, Scotland UK; 3 Academic Unit of Primary Medical Care, University of Sheffield, Sheffield, England UK; 4 Health Psychology, Radboud University Medical Centre, Radboud Institute of Health Sciences, IQ Healthcare, Nijmegen, Netherlands

**Keywords:** Cancer, Pain, Palliative care, Health informatics, Intervention mapping, Behaviour change

## Abstract

**Purpose:**

To develop a novel digital intervention to optimise cancer pain control in the community. This paper describes intervention development, content/rationale and initial feasibility testing.

**Methods:**

Determinants of suboptimal cancer pain management were characterised through two systematic reviews; patient, caregiver and healthcare professional (HCP) interviews (*n* = 39); and two HCP focus groups (*n* = 12). Intervention mapping was used to translate results into theory-based content, creating the app “Can-Pain”. Patients with/without a linked caregiver, their general practitioners and community palliative care nurses were recruited to feasibility test Can-Pain over 4 weeks.

**Results:**

Patients on strong opioids described challenges balancing pain levels with opioid intake, side effects and activities and communicating about pain management problems with HCPs. Can-Pain addresses these challenges through educational resources, contemporaneous short-acting opioid tracking and weekly patient-reported outcome monitoring. Novel aspects of Can-Pain include the use of contemporaneous breakthrough analgesic reports as a surrogate measure of pain control and measuring the level at which pain becomes bothersome to the individual.

Patients were unwell due to advanced cancer, making recruitment to feasibility testing difficult. Two patients and one caregiver used Can-Pain for 4 weeks, sharing weekly reports with four HCPs. Can-Pain highlighted unrecognised problems, promoted shared understanding about symptoms between patients and HCPs and supported shared decision-making.

**Conclusions:**

Preliminary testing suggests that Can-Pain is feasible and could promote patient-centred pain management. We will conduct further small-scale evaluations to inform a future randomised, stepped-wedge trial.

**Trial registration:**

Qualitative research: ClinicalTrials.gov, reference NCT02341846

Feasibility study: NIHR CPMS database ID 34172

**Electronic supplementary material:**

The online version of this article (10.1007/s00520-020-05510-0) contains supplementary material, which is available to authorized users.

## Background

Cancer incidence and prevalence rates are increasing [1]. Pain affects over a third of patients with cancer and over two thirds of patients with advanced cancer [2]. Cancer pain is distressing for patients and their families and is a frequent reason for hospital admissions and emergency department utilisation [3]. Pain is the most frequent reason for calls to out-of-hours primary care services by people with cancer [4, 5]. Individuals have difficulties communicating about pain, judging when to seek help and using prescribed analgesics effectively [6]. Pain and symptom control are achieved more often in inpatient and hospice settings than within the community [7].

There is increasing political interest in novel interventions that support individuals to be cared for safely, effectively and efficiently within the community [8, 9]. It has been proposed that digital technologies will increasingly support patients to communicate with the health service and to participate more actively in their care [9]. In oncology, digital technologies have been used to capture patient-reported outcome measures (PROMs) and to feed these back to healthcare professionals (HCPs) [10–12].

Two systematic reviews showed that PROM feedback can improve patient satisfaction with care and increase the number of symptoms discussed during consultations [13, 14]. Another review [15] found that PROM feedback interventions for cancer pain management reduce patient-reported pain intensity by approximately 1 point out of 10. The review highlighted problems with intervention fidelity and inadequate attention to how PROMs were integrated within clinical care to improve pain management [15].

Allsop et al. [16] reviewed information communication and technology systems designed for the identification, assessment or monitoring of pain in patients with cancer. Seventeen unique systems were identified. Twelve were for use by patients in clinic waiting rooms prior to appointments. Others collected PROMs by telephone via nurses or automated telephone lines. Only four systems allowed remote monitoring via Web-based forms, and no smartphone apps were identified [16]. Studies lacked detail on the rationale and development approaches taken and did not fully capitalise on the capabilities of digital technologies.

Digital interventions tend to have multiple interacting components [17]. The Medical Research Council (MRC) framework for complex intervention development stipulates that such interventions should identify and utilise existing evidence, theory and model processes and outcomes during the development phases [18].

The aim of this research was to develop a theory and evidence-based intervention to optimise cancer pain management in the community. The objectives were to fully understand the problem and to design an intervention that addressed the needs of those experiencing and managing cancer pain. This paper describes intervention development, intervention content and components, expected mechanisms of action and early feasibility testing.

## Methods

An intervention mapping (IM) approach guided this research project [19, 20]. IM is an established six-step, problem-based approach which allows behaviour change theory to be applied systematically to a health problem. The steps in IM are as follows: (1) modelling the problem, (2) specifying programme outcomes and objectives and creating a model of change, (3) programme design, (4) programme production, (5) creating a programme implementation plan and (6) planning evaluation. This paper deals with the first four steps.

### Step 1: modelling the problem

**Fig. 1:**
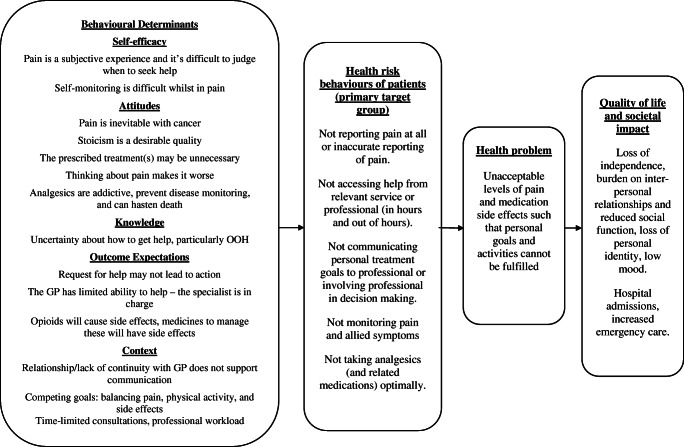
Logic model of patient behaviours and their determinants that can lead to unacceptable levels of pain and other negative outcomes

Step 1 involves fully characterising the problem and the behaviours involved in suboptimal cancer pain management. Existing literature was reviewed [15, 21], and interviews were conducted with patients with cancer pain (*n* = 14), their linked caregivers (*n* = 6) and HCPs (*n* = 19). Two multidisciplinary HCP focus groups were conducted [22]. Results of interviews and focus groups have previously been reported in detail [22]. Findings that influenced intervention content are summarised here, and a logic model of the problem is illustrated in Fig. 1.

Effective cancer pain management relies on multiple interacting patient and professional behaviours, including interpretation and reporting of pain by patients/accessing help, pain assessment/communicating about pain, analgesic prescribing and patients utilising analgesics optimally.

Patients experiencing problematic cancer pain tended to be prescribed strong opioids [22]. Breakthrough (short-acting “as-required” opioid) analgesic use was often considered to be a surrogate measure of pain control by professionals and was an important consideration during medical reviews. Concerns about opioids, mainly side effects and impact on function, were prominent in people with cancer pain. Patients made complex trade-offs between physical activity levels, pain intensity, analgesic side effects and social functioning in order to achieve individual goals, and did not always communicate these decisions to HCPs.

Managing cancer was highly burdensome for patients and their caregivers, and pain management was one of many competing considerations. A desirable intervention would add value to current management approaches without significantly adding to patient, caregiver or professional workload.

### Step 2: specifying programme outcomes and objectives and creating a model of change

The model of the problem derived in step 1 (Fig. 1) was used to identify a target population and to specify intervention goals, performance objectives (the relevant behaviours to be changed to achieve these goals) and determinants of target behaviours, for example knowledge, attitudes and self-efficacy. A logic model is provided (Online resource 1). The resulting intervention targets individuals prescribed strong opioids.

Intervention goals are to increase the proportion of patients who reported pain intensity levels within their *acceptable* range, to reduce the proportion reporting problems with opioid analgesics and to improve person-centred care and overall satisfaction with care. Performance objectives include the following: for patients and HCPs to discuss and agree treatment goals; for patients to monitor pain, important side effects, function and breakthrough opioid use; for patients to seek timely medical attention for unacceptable levels of pain; and for patients and community HCPs to review outcomes and adjust goals.

Behavioural change matrices (Online resource 2) were created, linking important and potentially changeable performance objectives with the behavioural determinants necessary to achieve the objective.

### Step 3: programme design

Creative ideas generated by all authors were assessed objectively against the specified performance objectives and behavioural determinants from step 2. Theoretical methods to change behaviour were chosen from published lists within IM [19]. Methods were selected according to the behavioural determinants to be changed, whilst considering which methods would fit best with practical intervention ideas. Methods underpinning the intervention include goal setting, self-monitoring of behaviour, tailoring and feedback. Further details of theoretical methods and how they fit within the intervention are presented in Table 1. The intervention has two main components: a digital app (Can-Pain) to be used by patients who are prescribed strong opioids, and a consultation with a HCP in which data generated by the app is used to give patients tailored feedback about pain management.

**Table 1:** App content, theoretical methods and expected mechanisms of action to improve pain management

App section and technological features	Content and/or educational messages	Examples of change objectives addressed (see Online resource 2)	Theoretical methods to change behaviour (from IM)	Rationale/mechanisms through which pain management could be improved
Breakthrough analgesic recording Tap screen to quickly record fast-acting opioid. The app times and dates the entry and adds it to the user’s weekly report screen Users receive a message asking them to seek help if pain is not improving in 30 min. An in-app algorithm automatically asks user to seek medical attention (and re-directs to a list of telephone help numbers) if 3 short-acting opioid doses are recorded in 24 h	User can quickly record fast-acting *breakthrough* opioids, and invited to record a pain trigger from a pre-defined list, including movement, stress and activities	Knowledge objective 9: Can explain important triggers for pain and how to manage these. Self-efficacy objective 9: Expresses confidence in judging when to seek help from professional	Self-monitoring Feedback Cue-altering (using the stimulus of breakthrough analgesic use to get the user to consider seeking early help for escalating/non-resolving pain) Facilitation (linking messages about seeking help to lists of telephone numbers to make seeking help easier)	Data on the number of short-acting doses is used by clinicians to inform long-acting opioid dose adjustments. Short-acting opioid dose can give insights into the adequacy of overall pain control. Patients can be reluctant to seek help, despite experiencing problematic pain—the app gives them specific cues to seek help, and directs them to a screen with useful telephone numbers
Weekly diary Touch-screen self-rating scales Computerised adaptive testing is used to tailor questions based on responses; e.g. reporting side effects leads to questions on the nature of side effects. These questions are skipped if side effect ratings are low. Diary report automatically emailed to pre-specified address at completion	Users self-rate pain (various dimensions) on a 0–10 point scale. A novel item asks about the level at which pain becomes bothersome. Site of pain can be indicated on an interactive body map, and word clouds contain descriptive terms, e.g. words that describe neuropathic pain, “pins and needles”, etc. Users are asked about mood, medication side effects, concerns about opioids, missed analgesic doses and reasons for missing doses. Users can enter free text information	Attitudes objective 2: Expresses the expectation that primary care professionals want to hear about personal treatment goals, and that the professional is able to assist in achieving these. Outcome expectation objective 6: Expects that the intermittent and event-triggered monitoring of pain, analgesic use and side effects can contribute to achieving treatment goals	Self-monitoring Tailoring (different questions based on characteristics of the participant, e.g. side effects, concerns about analgesics)	Reports are shared with clinicians to inform medical consultations and enhance pain assessment. The diary summarises the user’s current status with respect to pain/related symptom control, and whether users are experiencing levels of pain that are unacceptable to them. Output report is designed to promote discussion with clinicians about pain management expectations and any discrepancies between patient and professional goals and highlights any attitudinal barriers to analgesic utilisation which could be tackled by the clinician
View diary reports Natural language generation used to make bespoke reports from the diary and breakthrough entries, including visual summaries/graphs	Patients can view their breakthrough analgesic reports and weekly diary reports at any time	Self-efficacy objective 1: Is able to recognise and describe characteristics of their pain, exacerbating and relieving factors, triggers and personal response to analgesics Subjective norm objective 5: Expresses the expectation that disease and response to treatment can change (improve or deteriorate) over time and that pain management goals and plans may need to be adjusted	Feedback Consciousness raising	Insights into trends in pain control and triggers for pain/analgesic use could inform pain management approaches by the patient, e.g. taking an analgesic before a painful activity or recognising that stress/emotions are contributing to pain
Video about pain management	An actor represents a patient with cancer pain. The video depicts an interview between the actor (patient) and a GP. The patient discusses his fears about cancer pain, expectations about pain management and how he has overcome certain barriers to successful pain management. The patient and doctor discuss the nature of cancer pain, treatment options, using short- and long-acting opioids to control pain, how to manage side effects and problems that arise at night/weekends	Knowledge objective 3: Can explain different examples of how other patients like them balance pain, side effects and participation ability Outcome expectation objective 5: Expects that many side effects can be managed effectively Self-efficacy objective 6: Is able to plan for potential problems in the out-of-hours period and agree an action plan with community healthcare professional	Chunking—the video is in sections and has text descriptions at the end of each section Framing and persuasive communication—positive messages are used to persuade others to adopt optimal pain management approach Imagery—metaphors are used to aid understanding Information about others’ approval—the clinician emphasises that they expect to be contacted about pain management issues Modelling—patient (actor) is age appropriate with neutral accent and gives an example of how they controlled pain	Educational messages are directly derived from unmet patient needs elicited from qualitative enquiries with patients and existing literature
Useful Web links	Links to educational resources on pain and symptom management from reputable organisations	Knowledge objective 2: Can describe the available treatment options to control pain and their side effects	Facilitation	Sign-posting to existing educational resources that are kept up-to-date Improving knowledge about pain management techniques and treatment options could optimise self-management
Useful telephone numbers	These include out-of-hours medical contact numbers (Scotland) and the Macmillan nursing service. Users are also reminded to telephone their own medical practice during daytime hours	Knowledge objective 4: Knows who to contact in the community for assistance with symptom management	Facilitation	Qualitative interviews in the out-of-hours setting revealed that some patients did not know who to contact for help with pain control. This feature aims to facilitate access to medical care

### Step 4: programme production

A pictorial storyboard of Can-Pain was presented to a computer scientist. A mock version of the app was created using Microsoft PowerPoint and taken to healthy volunteers (health psychology students, academic colleagues, multidisciplinary clinicians and delegates at academic conferences). Individuals interacted with the mock app and offered verbal feedback, which was used to refine wording and presentation. Can-Pain was programmed using Ruby on Rails Web application framework. The current version requires Internet connectivity.

### Feasibility testing

A feasibility study was designed, in which data from four to six patients would be used to test usability, functionality, acceptability to patients/carers/clinicians and feasibility in clinical practice. Several of the planning group had experience of successful feasibility testing digital interventions with a small number of users [23, 24]. It was anticipated that four sets of linked patients, caregivers, nurses and doctors (i.e. 12 participants) would give substantial insights into the intervention experience, participant burden, acceptable duration and dose (e.g. frequency of diary entries, acceptability of diary length and questions, number of scheduled intervention consultations) and how the intervention would perform with respect to the behavioural targets identified during IM.

The World Health Organization suggest between 10 and 100 individuals should be involved in feasibility testing digital health interventions [25]. A more conservative sample size was selected because linked participants were being recruited together, and longitudinal data were being collected. Testing also involved a novel consultation model in a vulnerable patient group, and technical problems were anticipated during initial testing.

Recruitment to feasibility testing took place in four stages: first, Macmillan nurses (community palliative care nurses) were recruited by the research team via local networks. Second, the Macmillan nurse identified patients from their caseload who had cancer pain and were using/starting strong opioids. Third, the nurse approached the patient’s general practitioner (GP) to gauge interest in participation. Fourth, eligible patients of GPs who were interested in participating were given study invitation packs by their nurses and invited to reply directly to the research team if they wished to participate. Patients were asked to invite a caregiver to participate alongside them if they wished. Thus, Macmillan nurses, their linked patients and the patient’s linked GPs were recruited in triads, with or without a linked caregiver (at the discretion of the patient).

Patients were asked to use Can-Pain over a 4-week period. Can-Pain automatically logged patient breakthrough and diary entries and sent them immediately to a pre-programmed email address. During the study period, the reports were sent by email to the lead researcher, who forwarded data on a weekly basis to patients’ linked GP and Macmillan nurse. The Macmillan nurse was asked to schedule at least one clinical encounter with the patient.

The lead researcher (RA) gave participants a brief, user-led introduction to the app, and participants were provided with a Samsung Galaxy A7 tablet onto which the app had been loaded. Can-Pain was designed to be intuitive, but usability data were collected during feasibility testing to inform the need for additional training. Participants were given unique logins and passwords.

Patient participants were telephoned by the lead researcher each week to check for any problems and collect verbal feedback (brief telephone interview) about Can-Pain. An in-depth interview was performed at the end of the study with all patient/caregiver and professional participants. All interviews were conducted according to schedules. Brief interviews covered participants’ experiences of using the app and any problems experienced, particularly burden or technical issues. End-of-study patient/caregiver interviews probed experiences of using Can-Pain, any barriers to interacting with it and how the app influenced pain management, help-seeking and interactions with healthcare professionals. Professional interviews covered experiences of using the weekly pain/symptom reports, how they influenced care and pros/cons of integrating PROMS within clinical care. All participants were asked for suggestions about how to improve the intervention. All interviews were audio-recorded, transcribed verbatim and analysed using Framework and thematic analysis [26]. Quantitative data from app output reports and from user activity automatically logged by the app were analysed descriptively, and medical notes were reviewed to determine whether/how the intervention had been documented within episodes of primary care.

### Ethics

All participants gave informed consent to participate in qualitative research and feasibility testing. Approvals were granted by North of Scotland Regional ethics committee (qualitative research reference 15/NS/0002; feasibility testing reference 17/NS/0005) and NHS Research and Development.

## Results

### Can-Pain intervention content

**Fig. 2:**
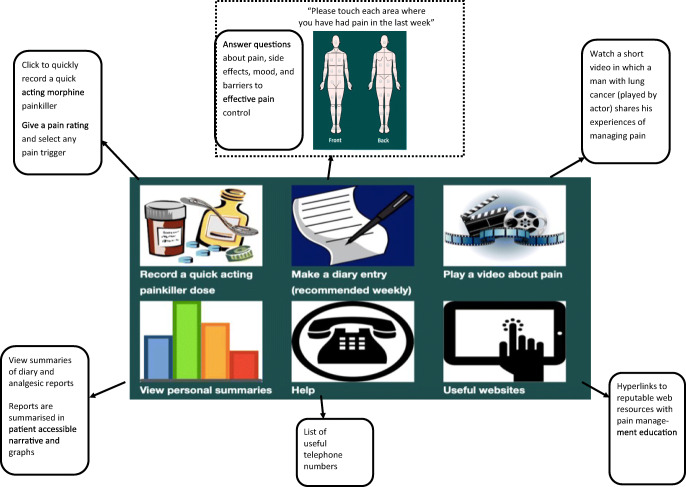
Annotated screenshot of Can-Pain dashboard

The Can-Pain app contains six sections accessible from a dashboard: breakthrough analgesic recording, a weekly diary, viewable summaries of previous diary and breakthrough reports, a video about pain management, useful Web links and telephone help. A screenshot of the dashboard is shown in Fig. 2. Intervention content, the theoretical methods employed and the rationale through which pain management could be improved are presented in detail in Table 1.

### Feasibility testing

#### Recruitment and retention

Seven Macmillan nurses were recruited by the research team. Of these, two nurses recruited two patients, one linked caregiver and two linked GPs. Five nurses who did not recruit a patient/linked GP took no further part in the study, but all other participants completed the full study.

Patient recruitment was challenging, and delays in app programming and hosting on the secure server reduced time available (within our time-limited project) for feasibility testing from 6 to 4 months. Macmillan nurses had minimal contact with patients who were stable. Nurses were not asked to record patients to whom they informally mentioned the study but noted that unpredictable or rapidly deteriorating health status and admission to hospital were significant barriers to recruitment. Nurses also reported not inviting patients whom they judged might be uninterested in digital technology or the intervention. One patient returned his reply slip and had a GP who consented to participate but deteriorated clinically such that he was unable to participate.

#### Patient characteristics and their reported pain data

The demographics of both patient participants are presented in Table 2. Both had bony metastases and were on a combination of long- and short-acting strong opioids and a gabapentinoid.

**Table 2:** Patient participant demographics in feasibility study

Patient number, sex	Cancer diagnosis	Age	SIMD 2012 decile [43]*	Urban-rural 6-fold category [44]	Analgesic regime at enrolment	Caregiver participant	Overall pain rating at baseline**
Patient 1, female	Metastatic myeloma	73	4	1 (large urban)	Twice daily modified-release hydromorphone, as required immediate-release hydromorphone, regular gabapentin	Yes, male partner	6
Patient 2, male	Renal cancer with bony metastases	55	8	1 (large urban)	Twice daily modified-release OxyContin, as required immediate-release OxyContin, regular pregabalin	No	5

*Scottish Index of Multiple Deprivation (SIMD) 2012 ranks areas in Scotland by postcode from 1 (most deprived) to 6505 (least deprived) according to multiple indicators of deprivation such as employment and housing. Ranks are reported here by decile with 1 indicating most deprived and 10 indicating least deprived

**In-app self-rating where 0 is anchored “no pain” and 10 is anchored “pain as bad as I can imagine”

Patient 1 had little variation in her self-reported pain and side effect ratings over the study period, rating overall pain levels between 6 and 7 out of 10, pain becoming *bothersome* between 5 and 7 out of 10 and analgesic side effects between 5 and 7 out of 10. She used breakthrough analgesia twice to three times every day, routinely taking a breakthrough dose around 11 pm.

Patient 2 also had stable pain ratings, with overall pain between 4 and 5 points out of 10, and reported considering pain bothersome at 6 out of 10. His pain ratings never crossed this threshold. He used on average three breakthrough opioid doses per week. Stress, movement and activity were pain triggers.

#### Insights about the intervention from qualitative interviews

Qualitative interviews with all participants (*n* = 7) (two GPs, two nurses [one interview each], two patients/one caregiver [four longitudinal interviews each], caregiver/patient [joint interviews]) generated around 4 hours of audio-recorded interview data over 4 weeks. Weekly telephone interviews with patients/caregiver had an average duration of 15 min. End-of-study interviews with patients/caregiver had an average duration of 38 min, with HCP interviews averaging 17 min.

Interviews gave insights into advantages of the intervention, limitations, engagement and usability, technical issues and suggestions for further development.

Patient/caregiver participants felt that being closely monitored was an advantage and judged that their monitoring reports would help their linked HCPs to effectively prioritise their caseload and recognise problems if they arose. They also observed that longitudinal symptom data might be more meaningful to HCPs than assessments at a single point.

Patient 1’s reports of consistently high pain scores led to discussions with her HCPs about increasing her analgesic dose, which she was not keen to do. The perceived discordance between problematic pain and patient reluctance to increase analgesia led her professionals to explore the reasons for this, including any concerns about strong opioids.

“What she’s recording there is that she’s quite plainly sore a lot of the time and the quality of it, you know, she’s not happy with being that sore, but then when you actually speak to her (…) “I don’t really want to increase my painkillers”. And we would explore you know, are you worried about them or anything like that, and not really, (…) I think actually what’s going on there is perhaps a larger thing about how she thinks about her illness (…) it’s caught up a bit more in her coping mechanisms”. (Patient 1’s GP)

Patient 2 logged low mood in his diary and reported that stress was a trigger for breakthrough analgesic use, explaining in his study interview that his emotions strongly influenced his perception of pain, but that doctors were more interested in *hard facts* during time-limited consultations. Reports of patient 2 were used by his GP to start conversations about mood and stress.

“I could easily identify what causes, what triggers him to take a breakthrough, (…) which in his case was mainly stress and it also really highlighted, which perhaps we hadn’t identified just quite how he was feeling in himself about the low mood and feeling hopeless at times and worthless, which I was able to use as a cue to discuss those feelings in more detail with him, so I thought that was really beneficial”. (Patient 2’s GP)

These conversations and references to the app reports were evident in the electronic medical record.

None of the patient or professional participants found the intervention burdensome or onerous. Patient 1’s caregiver took charge of app administration, logging her breakthrough doses, asking her to rate pain, reading diary questions to her and entering data on her behalf. The app seemed to be a natural extension to the roles and tasks that he had already adopted as a caregiver.

The main limitation of the intervention from a patient/caregiver perspective was difficulty summarising a complex phenomenon like pain within a diary that utilised numerical ratings. Word clouds with qualitative descriptions of the pain, and the body map for pain location, helped to an extent, but patients pointed out that several types of pain could co-exist and were difficult to summarise.

There were technical issues during feasibility testing: the app ran slowly at times, there were issues with screen sizing, there were two episodes of a patient being routed to a blank screen after logging an analgesic dose and predictive text features were found to be *fiddly*. Participants gave suggestions for additional triggers for breakthrough pain that might be included in the next version of the app. Participants would also have liked the ability to log breakthrough doses retrospectively.

App usage data are presented (Online data source 3). Patient 1/her caregiver logged 62 breakthrough doses over the study period whilst patient 2 logged 13. All sections of the app were utilised over the study period except for the list of telephone help numbers, which was not used by patient 2.

## Discussion

### Main findings

Patients, their caregivers and HCPs face multiple challenges when managing cancer pain. Can-Pain has been created to support important self-management behaviours. There are early indications that the app is feasible and acceptable to patients, caregivers and HCPs. A key component is feedback of patient-reported data between patients and HCPs. To the best of our knowledge, this is the first digital intervention to use contemporaneous breakthrough analgesic reports as a surrogate measure of pain control and to measure the level at which pain becomes bothersome to the individual. This acknowledges that some patients accept a certain amount of pain and make trade-offs with other important social and functional activities. HCPs used patient reports intuitively to explore patient experiences and treatment goals in depth and to rule out misconceptions about analgesics or their underutilisation.

### Context with other literature

A scoping review of publicly available apps for cancer survivors found that many apps offered symptom tracking and graphing capabilities along with educational information about cancer [27]. None focused specifically on cancer pain. Most were developed by commercial organisations, and some contained content that was potentially exploitative (e.g. selling *cures* for cancer). Other recognised problems with healthcare apps include lack of scientific/clinician input into content and failure to involve patients in design [28]. Failure to involve patients or to consider complexity can result in technology that does not address important real-world clinical problems, and lack of adoption [29, 30].

Other digital interventions exist that support PROM feedback for individuals with symptomatic cancer [10, 12, 31–33]. Some provide Web-based pain management advice [34], deliver psychological therapies or support for individuals with cancer pain [35, 36] or focus on specific situations such as post-surgical pain management [37]. Most psycho-educational and PROM feedback interventions can achieve small reductions in pain intensity, and it is difficult to know which components are effective [21].

### Strengths, limitations and issues still to be established

Patients and clinicians were involved in intervention development. Intervention components were selected based on behavioural principles, supported by behavioural theories. This should make Can-Pain easy to replicate and protect core intervention components from becoming outdated as technology evolves [38].

The target population for Can-Pain is at risk of unpredictable deterioration and is difficult to recruit into clinical research [39–41]. Our feasibility study design contributed to recruitment difficulties. We relied upon busy nurses to recruit patients and their linked GPs. The multistep recruitment process added complexity. Furthermore, nurses’ main clinical workload involved patients who were deteriorating. They had less contact with well patients.

In feasibility testing, both patients had stable pain due to bony metastases, were white Scottish, lived in urban environments and were on similar analgesic regimens. It will be essential to gather further data about how Can-Pain performs in patients with diverse demographic characteristics. Preliminary findings also suggest that caregivers play a key role in supporting loved ones to manage cancer and pain management. In this study, caregivers were recruited optionally via patients. An alternative strategy would be to recruit caregivers directly, via cancer support organisations for example. Further research is required to fully delineate the role of caregivers in promoting engagement with the intervention and whether caregivers influence the PROM data collected.

### Next steps

A commercial partner will be engaged to optimise the app technically and to make it compatible with major app stores. In eHealth research, there is an argument that software should be continually improved and updated based on user feedback and that every version is a “beta version” [38]. This can make traditional randomised controlled trials impractical. In the next phases of research, collaborations will be formed with hospices, third-sector organisations and researchers in other geographical settings to perform multiple small-scale evaluations. Taken together, these will give major insights into usability and feasibility of Can-Pain and help to establish the most important outcome measures for a future trial.

Outcomes will likely relate to patient-centred care, perceived control and satisfaction. It could also be possible to embed short, validated measurements of pain, quality of life and performance status into the intervention. Numerical 0 to 10-point pain rating scales were unpopular with both patients who took part in feasibility testing, whereas descriptive words were more intuitive. It would be prudent to consider embedding categorical measures of pain with verbal descriptors in addition to numerical ratings.

*Burden* was a major theme in formative research. Treatment burden (the workload of healthcare and its impact on patient function) is emerging as a major concern globally, particularly for individuals with multimorbidity. There are now several validated measurement tools to assess treatment burden (for example, a 10-item scale by Duncan et al. [42]) which could provide important insights into any added burden of embedding Can-Pain into routine care.

Ultimately, it will be important to test the efficacy and cost-effectiveness of Can-Pain. An advantage of digital technology is that it is rapidly scalable. A randomised step-wedged implementation trial with embedded economic evaluation could be an efficient design through which Can-Pain could be simultaneously implemented and evaluated in the community.

## Conclusion

Can-Pain has been designed systematically with input from key stakeholders. Core components are underpinned by theories from behavioural science. Can-Pain could be a promising way of using PROMs to enhance the management of patients with symptomatic cancer within the community setting. We anticipate that Can-Pain could help professionals to recognise problems and could help patients and professionals communicate efficiently about subtler aspects of pain control, without causing unacceptable burden. We will design the next stages of testing to take account of a target population who are seriously unwell and to exploit the accessible and scalable nature of digital technology.

## Electronic supplementary material

ESM 1 (DOCX 21.5 kb)

ESM 2 (DOCX 15.6 kb)

ESM 3 (DOCX 66.9 kb)
