# Supplementary material for: Can-Pain-a digital intervention to optimise cancer pain control in the community: development and feasibility testing
Source: Support Care Cancer. 2020 May 28;29(2):759–69. doi: 10.1007/s00520-020-05510-0 (PMC7767903; doi:10.1007/s00520-020-05510-0)
Supplement: Supplementary file 1 — (DOCX 21.5 kb) [file 520_2020_5510_MOESM1_ESM.docx]

Supplementary Data File 1: A logic model of change for individuals with cancer pain

**Personal determinants**

Knowledge

Attitudes

Outcome expectations

Self-efficacy

Skills

Subjective norms

**Performance objectives**

1. Set treatment goals based on own preferences for pain management, side effects, and ability to participate in daily activities
2. Communicate own treatment goals to community healthcare professional, balancing side effects, analgesic use, pain, and functional goals
3. Seek medical attention timeously (in hours, and out of hours) for unacceptable levels of pain
4. Adhere to agreed management regime in terms of adherence to regular medication, using breakthrough analgesics when required, and moderating daily activities to keep pain levels manageable
5. Monitor pain, important side effects, and functioning on a weekly basis and record breakthrough analgesic use as it is taken.
6. Review outcomes and adjust goals with community healthcare professional.

.

**Behavioural outcomes**

Reports pain accurately and timeously

Monitors pain and allied symptoms

Communicates about personal treatment goals and pain management experiences/problems to the professional

Takes analgesics and related medication optimally

**Health**

Acceptable levels of pain intensity and physical activity

Reduced levels of analgesic side effects

**Quality of life and societal**

Improved mood, improved social function.

Appropriate and timeous use of medical care, reduced emergency care
