# Supplementary material for: Can-Pain-a digital intervention to optimise cancer pain control in the community: development and feasibility testing
Source: Support Care Cancer. 2020 May 28;29(2):759–69. doi: 10.1007/s00520-020-05510-0 (PMC7767903; doi:10.1007/s00520-020-05510-0)
Supplement: Supplementary file 2 — (DOCX 15.6 kb) [file 520_2020_5510_MOESM2_ESM.docx]

**Supplementary Data File 2: Patient Behavioural Change Matrix**

| **Personal Determinants** | **Knowledge (K)** | **Attitudes (A) and Outcome Expectations (OE)** | **Self-efficacy and skills (S)** | **Subjective Norms (SN)** |
| --- | --- | --- | --- | --- |
| **Performance Objectives (PO)** |  |  |  |  |
| PO1: Set treatment goals based on own preferences for pain management, side effects, and ability to participate in daily activities | **K1:** Can explain the nature of cancer pain, potential causes, and knows that pain is not necessarily a useful marker of disease activity  **K2:** Can describe the available treatment options to control pain and their side effects  **K3**: Can explain different examples of how other patients like them balance pain, side effects, and participation ability | **A1:** States that it is important to clearly define, and agree with HCP and family, personal goals for pain management, function, symptom control and side effects, given personal lifestyle and preferences  **OE1:** Expects that good pain management and functional goals can be achieved | **SE1:** Is able to recognize and describe characteristics of their pain, exacerbating and relieving factors, triggers, and personal response to analgesics.  **SE2:** Is able to consider and decide upon personal priorities with respect to lifestyle, function, and symptom/side-effect control  **SE3:** Is able to articulate realistic functional goals  **SE4:** Is able to explain how functional goals may be achieved by balancing pain, medication use, and physical activity | **SN1:** States that their HCP expects them to express their preferences, so that treatment and support can be tailored |
| PO2: Communicate own treatment goals to community healthcare professional, balancing side effects, analgesic use, pain, and functional goals | **K4:** Knows who to contact in the community for assistance with symptom management.  **K5:** Knows how and when to access appropriate community health professionals | **A2:** Expresses the expectation that primary care professionals want to hear their own treatment goals, and that they are able to assist in achieving these | **SE5:** Expresses confidence in own ability to clearly discuss treatment goals with professional | **SN2:** Expresses the expectation that HCPs will respect patient treatment preferences and assist in achieving these through available means |
| **Personal Determinants** | **Knowledge (K)** | **Attitudes (A) and Outcome Expectations (OE)** | **Self-efficacy and skills (S)** | **Subjective Norms (SN)** |
| **Performance Objectives (PO)** |  |  |  |  |
| PO3: Seek medical attention timeously (in-hours and out of hours) for unacceptable levels of pain | **K6:** Knows how to contact out of hours service and under which circumstances  **K7:** Describes the benefits of planning ahead for unexpected worsening of symptoms | **OE2:** Recognises that there is capacity within the health system to deal with unexpected symptoms | **SE6:** Is able to plan for potential problems in the out of hours period and agree an action plan with community health professional | **SN3:** Expresses the expectation that medical professionals wish to be informed about unacceptable levels of pain and associated symptoms |
| PO4: Adhere to agreed management regime in terms of adherence to regular medication, using breakthrough analgesics when required, and moderating daily activities to keep pain levels manageable | **K8:** Can describe the rationale for regular analgesic use and breakthrough analgesic use and the importance of using long-acting analgesics at set intervals.  **K9:** Can explain important triggers for pain and how to manage these | **A3:** Believes that addiction is rarely a problem in cancer pain management  **A4:** Recognises that opioid analgesics are generally safe  **A5:** Recognises that the use of opioid analgesics as prescribed to control pain will not hasten death  **OE3:** Expects that the regular use of analgesics will contribute to better controlled pain, prevention of breakthrough pain, and achievement of functional goals.  **OE4:** Expects that moderating exposure to pain triggers will lead to overall better functioning  **OE5:** Expects that many side effects can be managed effectively | **SE7**: Is able to take long acting analgesics at regular intervals  **SE8:** Expresses confidence in own ability to manage breakthrough pain using short-acting medication | **SN4:** Feels supported by caregiver/ treatment supporter and HCP in achieving functional and pain goals through medication use and managing pain triggers |
| **Personal Determinants** | **Knowledge (K)** | **Attitudes (A) and Outcome Expectations (OE)** | **Self-efficacy and skills (S)** | **Subjective Norms (SN)** |
| **Performance Objectives**  **(PO)** |  |  |  |  |
| PO5: Monitor pain, important side effects, and functioning on a weekly basis and records breakthrough use | **K10:** Can explain how and when to monitor pain, side-effects, analgesic use, and functioning | **OE6:** Expects that the intermittent and event-triggered monitoring of pain, analgesic use and side effects can contribute to achieving treatment goals | **SE9:** Expresses confidence in judging when to seek help from professional |  |
| PO6: Review outcomes and adjust goals with community healthcare professional | **K11:** knows that disease and response to treatment can change (improve or deteriorate) over time and that pain management goals and plans may need to be adjusted | **OE7:** Expects that goals and management plan will be informed by self-reports of symptoms, side effects, and function | **SE10:** Is able to articulate changes in symptoms and function, and to regularly re-visit personal goals and management strategies with health care professional |  |
