# Supplementary material for: Can-Pain-a digital intervention to optimise cancer pain control in the community: development and feasibility testing
Source: Support Care Cancer. 2020 May 28;29(2):759–69. doi: 10.1007/s00520-020-05510-0 (PMC7767903; doi:10.1007/s00520-020-05510-0)
Supplement: Supplementary file 3 — (DOCX 66.9 kb) [file 520_2020_5510_MOESM3_ESM.docx]

**Online data source 3: App usage reports**

| **Interactions per week** | Number logged week 1 | | Number logged week 2 | | Number logged week 3 | | Number logged week 4 | | Total logged over study period | |
| --- | --- | --- | --- | --- | --- | --- | --- | --- | --- | --- |
| **App feature** |  |  |  |  |  |  |  |  |  |  |
|  | **P1*** | **P2*** | **P1** | **P2** | **P1** | **P2** | **P1** | **P2** | **P1** | **P2** |
| Breakthrough tracking | 15 | 3 | 15 | 3 | 18 | 2 | 14 | 5 | 62 | 13 |
| Weekly diary completion | 1 | 1 | 1 | 1 | 1 | 1 | 1 | 1 | 4 | 4 |
| Views of breakthrough dose summary | 2 | 2 | 3 | 8 | 0 | 1 | 0 | 4 | 5 | 15 |
| View of weekly diary summary | 1 | 0 | 4 | 2 | 0 | 1 | 0 | 1 | 5 | 4 |
| Views of short film | 0 | 0 | 0 | 0 | 0 | 1 | 1 | 1 | 1 | 2 |
| Views of telephone help numbers | 1 | 0 | 3 | 0 | 0 | 0 | 0 | 0 | 4 | 0 |
| Views of web links | 3 | 2 | 1 | 0 | 0 | 0 | 0 | 1 | 4 | 3 |

*****P1 = Patient one, P2 = Patient 2
